# Neuroprotective effect of salvianolate lyophilized injection against cerebral ischemia in type 1 diabetic rats

**DOI:** 10.1186/s12906-017-1738-8

**Published:** 2017-05-10

**Authors:** Fujiang Wang, Qiansong He, Jinxin Wang, Qing Yuan, Hong Guo, Lijuan Chai, Shaoxia Wang, Limin Hu, Yue Zhang

**Affiliations:** 1 Tianjin State Key Laboratory of Modern Chinese Medicine, Tianjin University of Traditional Chinese Medicine, 312 Anshanxi Road, Nankai District, Tianjin, 300193 China; 2 Guiyang College of Traditional Chinese Medicine, Guiyang, 550025 People’s Republic of China; 3 Key Laboratory of Formula of Traditional Chinese Medicine (Tianjin University of Traditional Chinese Medicine), Ministry of Education, Tianjin, 300193 China

**Keywords:** Stroke, Diabetes, Salvianolate lyophilized injection, Nrf2/HO-1 pathway

## Abstract

**Background:**

Salvianolate lyophilized injection (SLI) has been clinically used in China for the treatment of acutely cerebral infarction. Clinical and experimental studies have shown that Diabetes mellitus (DM) not only increases the risk of ischemic stroke recurrence but also leads to poor outcomes and increases fatality rates after stroke. Our previous study has proved that SLI can reduce the infarct volume after stroke in type 1 diabetic rats. The aim of the study is to explore the mechanism of SLI on stroke outcome in type 1 diabetic (T1DM) rats.

**Methods:**

Type 1 diabetes rats model (T1DM) was induced in male Wistar rats by intraperitoneal (i.p) injection of streptozotocin (60 mg/kg) and T1DM rats were subjected to intraluminal middle cerebral artery occlusion (MCAO). The T1DM + MCAO rats were randomly divided into six groups: sham-operated, model-vehicle, positive control group (Edaravone-treating, DE 6 mg/kg) and SLI-treating group (10.5 mg/kg, 21 mg/kg and 42 mg/kg). SLI and DE were administered by tail vein injection at 3 h after MCAO, then daily for 14 days. Micro-CT scans of the brain tissue revealed vessel characteristics and distribution in the ischemia zone. Glucose uptake was analyzed by PET/CT. RAGE, MMP9 and inflammatory factors (COX-2, TNF-α and ICAM-1), HQ-1, HQO-1 and Nrf-2 expression levels in the ischemic brain tissue were analyzed by Immunofluorescence staining and Western blot at 14 days after MCAO.

**Results:**

In this study, we have demonstrated that SLI treatment significantly increased the number of brain microvasculature in ipsilateral and glucose uptake in cortex, hippocampus and penumbra in the T1DM + MCAO rats. SLI also significantly decreased the expression of RAGE, MMP9 and inflammatory factors expression, and increased the expression of HQ-1, HQO-1 and Nrf-2 in T1DM + MCAO rats.

**Conclusion:**

The study showed that SLI could protect against cerebral ischemia injury in T1DM + MCAO rats and the mechanism is related to decrease inflammatory factors and activate of the Nrf2/HO-1 signaling pathway.

## Background

Stroke is the second leading risk factor for causing death and the leading cause of long-term disability worldwide [1]. Diabetes mellitus (DM) is an independent risk factor for ischemic stroke. Notably, it affects a growing proportion of the population and is a major risk factor for stroke patients [2, 3]. About 30% of stroke patients are diabetics, and more than 50% of them develop towards post-stroke hyperglycemia [4]. Clinically, hyperglycemia may account for poor prognosis after ischemic stroke. Diabetes is also associated with higher mortality, more severe disability and a higher frequency of recurrent stroke [5]. Thus, there is an urgent unmet medical need for an effective novel therapy for stroke patients with diabetes. Advanced Glycation End Products (AGEs) play an important role in the cause and development of diabetic complications such as cardiovascular disease, stroke and other diseases [6]. RAGE activates to neural injury following cerebral ischemia in RAGE-targeted transgenic mouse, which indicates that RAGE directly contributes to pathology in cerebral ischemia [7]. The pro-inflammatory cytokines such as tumor necrosis factor (TNF-α) and intercellular adhesion molecule-1 (ICAM-1) may aggravate infarction, brain edema and neuronal death [8]. Nuclear factor erythroid 2-related factor 2 (Nrf2) protects cells against oxidative stress, and activates the transcription of antioxidant stress genes, including heme-oxygenase-1 (HO-1). The Nrf2/HO-1 pathway plays an important neuroprotective role in brain injury after ischemic stroke [9].

Danshen (Salvia miltiorrhiza), the dried root of Salvia miltiorrhiza, is a very famous Traditional Chinese medicine, which has been widely used in China, to a lesser extent in other oriental countries for treatment of cardiovascular and cerebrovascular diseases for thousands of years [4, 10, 11]. Salvianolate lyophilized injection (SLI), composed of the Salvia miltiorrhiza aqueous extraction (mainly including salvianolic acid B, salvianolic acid E, lithospermic acid and rosmarinic acid), has been approved in the treatment of stroke by the State Food and Drug Administration in China since 2011 [10]. In the pharmacological and clinical studies, these compounds were found to be strong antioxidants and potent free radical scavengers and could improve blood circulation, reduce the area of cerebral infarct, and inhibit the renin angiotensin system [12–14]. Research has shown that the composition of salvia miltiorrhiza has protective effects against focal cerebral ischemia/reperfusion injury [15], and there is no report on the effect of the active components in ischemic brain injury in type 1 diabetic rats.

We previously reported that SLI can improve functional recovery after stroke in diabetic rats [4]. In the present study, we demonstrated the neuro-protective effects of SLI against focal cerebral ischemia/reperfusion injury in type 1 diabetic rat model. And its mechanism may be related with multiple mechanisms of action, including decreasing the expression of RAGE, MMP9 and inflammatory factors and up-regulating the Nrf2/HO-1 antioxidant pathway.

## Methods

### Animal

Male wistar rats (250–280 g body weight) were purchased from Vital River Laboratory Animal Technology Co., Ltd, (Certificate no: SCXK Jing 2012-0001), kept in a 12-h dark/light cycle in a temperature 22 ± 2 °C and humidity 40 ± 5%, and fed on standard laboratory diet and water ad libitum. This study was carried out in strict accordance with the recommendations in the Guidance Suggestions for the Care and Use of Laboratory Animals issued by the Ministry of Science and Technology of China. Each rat was only used once. The experimental procedures followed the European Union (EU) adopted Directive 2010/63/EU. All animals were handled according to the guidelines of Tianjin University of Traditional Chinese Medicine (TCM) Animal Research Committee (TCM-LAEC2015028).

### Drugs and reagents

**Fig. 1:**
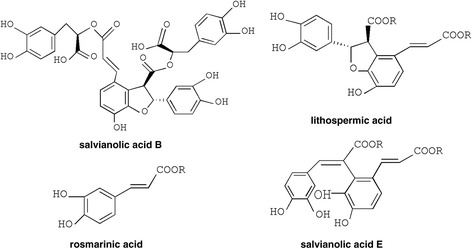
Chemical structures of main constituents in SLI

Salvianolate lyophilized injection (SLI), provided by Tianjin tasly Pharmaceutical Co., Ltd. (Tianjin, China), was authenticated and standardized in accordance with the Pharmacopoeia of China 2011. The dried root of Salvia miltiorrhiza was extracted by 80% ethanol under reflux for 3 times (3 h per time). The extracting solution was merged for decompressing concentration till there was no alcohol taste. The 80% ethanol extract was dried to obtain SLI. SLI were multiple salvianolic acids mainly including salvianolic acid B (SalB) (62.1%), salvianolic acid E (SalE) (1.8%), lithospermic acid (LA) (3.7%) and rosmarinic acid (RA) (4.5%) [10, 16]. The structures of these constituents are shown in Fig. 1. Edaravone (Batch number: H20031342) was purchased from Nanjing Simcere Pharma Co., Ltd. (Jiangsu, China).

Chloral hydrate (Batch number: Q/12HB 4218-2009) was purchased from Tianjin Kermel Chemical Reagent Co., Ltd. (Tianjin, China), freshly prepared to 3.5% solution with saline before experiment. Rabbit-anti-rat monoclonal antibodies for Heme-oxygenase-1 (HO-1), NAD (P) H quinine oxidoreductase (HQO-1), Nuclear factor erythroid 2-related factor 2 (Nrf2), Matrix metallopeptidases 9 (MMP9) and Cyclo-oxyge-nase-2 (COX-2) were purchased from Santa Cruz. (USA). Antibodies for Tumor necrosis factor-$\boldsymbol{\upalpha}$ (TNF-α) and Intercellular cell adhesion molecule-1 (ICAM-1) were purchased from Abcam Inc.(USA) and Receptor for Advanced Glycation End Products (RAGE) was purchased from Wuhan Boster Biological Technology, Ltd. (Wuhan, China). Secondary antibodies were purchased from Zhongshan Golden Bridge Biotechnology Co., Ltd. (Beijing, China). HE Staining Kit was purchased from Wuhan Boster Biological Co., Ltd. (Wuhan, China).

### Induction of type I diabetes in rats

Diabetes was induced by a single intraperitoneal injection of streptozotocin (STZ, Sigma, St. Louis, MO, USA) (60 mg/kg dissolved in sodium citrate, 0.1 mM, pH4.5) to adult male rats, as previously described [17]. The fasting blood glucose level from a tail vein sample was tested by using a glucose analyzer (ONETOUCH Ultra System, Johnson & Johnson, USA). Diabetes was defined by fasting blood glucose >15 mmol/L, Animals were subjected to stroke 2 weeks after diabetes induction.

### Focal cerebral ischemia-reperfusion injury model

Focal cerebral ischemia-reperfusion was induced with a minor modification of intraluminal middle cerebral artery occlusion (MCAO) as described previously [18, 19]. Briefly, T1DM rats were initially anesthetized with 4% isoflurane during induction and then maintained with 2% isoflurane in a mixture of 30% O_2_ and 70% N_2_O. Body temperature was monitored and maintained at 37 °C using a feedback-regulated water heating system. Under the operating microscope, the right common carotid artery (CCA), external carotid artery (ECA) and internal carotid artery (ICA) were separated via a cervical midline incision. A 3-0 monofilament nylon suture (Beijing Shadong Biological Technology Co., Ltd., Beijing, China) was introduced into the ECA lumen and extended into the ICA (18.5 ± 0.5 mm) to block the origin of the MCA. The suture was advanced into the ICA until MCA flow was <30% of baseline in order to exclude incomplete ischemia. The focal cerebral ischemia was confirmed by obvious changes of regional cerebral blood flow on laser Doppler perfusion monitor (PeriFlux System 5010, Perimed, Stockholm, Sweden). After 1.5 h of ischemia, the nylon suture was removed to establish reperfusion. After arousal from anesthesia, the rats were returned to their cages with ad libitum access to food and water. Sham-operated animals were subjected to the same surgical procedure, but the suture was not advanced beyond the internal carotid bifurcation.

Adequacy of anesthesia was controlled by monitoring corneal reflex and the lack of response to toe-pinching. Euthanasia was performed by excessive inhalation of isoflurane. Death was monitored by the cardiac activity and respiration. All efforts were made to minimize animal suffering and to reduce the number of animals used.

### Treatment groups and drug administration

Salvianolate Lyophilized Injection (SLI) was freshly dissolved in normal saline before use. Rats were randomly divided into six groups: sham-operated (DM), MCAO model (DM + MCAO), Edaravone (as a positive control drug)6 mg/kg (DM + MCAO + DE) and SLI 5.25 mg/kg (DM + MCAO + SLI-5.25), SLI 10.5 mg/kg (DM + MCAO + SLI-10.5), SLI 21 mg/kg (DM + MCAO + SLI-21), 10.5 mg/kg dose was converted from a commonly used dosage of SLI (200 mg/day) in clinical practice in our study. The rats were administrated Edaravone (6 mg/kg) or SLI (5.25, 10.5, 21 mg/kg) initially by intravenous injection (i.v) 3 h after the induction of transient MCAO and then daily for 14 days. The sham-operated group and MCAO model group were treated with isodose saline. In all experiments, data were collected by a blinded, randomized and controlled design. Number of animals in each group for determination of each parameter (See Table 1 for detail).

**Table 1:** Number of animals for different experimental groups and various parameters at 14 day after administration

	sham-op erated group	MCAO model group	Edaravone group	SLI 5.25 mg/kg group	SLI 10.5 mg/kg group	SLI 21 mg/kg group	total
HE staining	3	3	3	0	3	3	15
Micro-CT	4	4	0	0	0	4	12
PET-CT	0	4	0	0	0	4	8
Western blot analysis	6	6	6	6	6	6	36
immunofluorescence	6	6	6	6	6	6	36
total	19	23	15	12	15	23	107

### Hematoxylin-eosin staining (HE staining)

After 14 days of reperfusion, the rats were perfusion-fixed with 4% paraformaldehyde in 0.1 M phosphate buffer (pH7.4) under anesthesia. The paraffin-embedded brain sections (5 μm) were prepared and stained with hematoxylin and eosin. Histological evaluations were performed with HE-staining for assessing neuronal damage in the penumbra of ischemic cortex. In order to observe cell morphology, the pathological sections were observed under light microscopy (Leica Microsystems) at 400 × amplified.

### Micro-CT scans

Micro-CT scans were performed at 14 days, rats were anesthetized with chloral hydrate (3.5%, peritoneal injection, 10.5 mg/kg for maintenance during surgery) after 30 min give drugs, and perfused through the heart with heparin saline (500iu/ml) 500 ml, then fixed 15 min with 4% paraformaldehyde, after 15 min perfused Microfilm and 4 °C overnight. Brain tissue were dehydrated through a series of graded alcohol (50%,75%,85%,95%,100%), The micro-CT (QuantumFXuCT PerkinElmer,USA) scans of the brain were reconstructed to show the vessels (Fig. 4). Scans for all staining assays were performed using the following settings: 65 kV at 80 μA, resolution ratio (12.149 × 12.149 × 12.149) μm^3^ and exposure time of 2.96 ms to provide a suitable noise to signal ratio. Micro-view software was used to reconstruction 3D vessel image and calculated Connectivity Density (Conn.D), Mean Vascular Separation (M.Sp), Mean Vascular Thickness (M,Th) and Vascular Volume Fraction (VVF).

### PET/CT acquisition

After overnight fast, the rats were injected approximately 1 mCi/kg of 18 F-deoxyglucose (18 F-FDG, Sigam, USA) of rats into tail vein 1 h before PET acquisition. Rats were anesthetized using 3.5% isofluorane and maintained by 1.5% of isofluorane in 95% O_2_ and 5%CO_2_ approximately 40 min after 18 F-FDG injection. Anesthetized rats were placed supine on a heated bed in the gantry of an Inveon (Siemens Knoxville, TN) preclinical PET/CT/SPECT (Inveon Acquisition Workplace, version 1.5). Micro-CT Scan (Voltage: 80KV, Current: 30 mA, Slice thickness 2 mm, Echo times: 0.8 s), focused on the brain, was captured before acquisition PET imaging (Scan duration 15 min). PET images were co-registered to CT data (PFUSION tool, PMOD Technologies, Zurich, Switzerland). Each acquired PET image consisted of a 128 × 128 × 159 matrix at a voxel size of 0.8 × 0.8 × 0.8 mm; CT image, a 1024 × 1024 × 1024 matrix at a voxel size of 0.1 × 0.1 × 0.1 mm.We calculated the mean standardized uptake value (SUV) for ischemic side cortex, hippocampus and penumbra. The SUV value = the image derived radioactivity concentration/the whole body concentration of the injected radioactivity.

### Immunofluorescence

The rats were deeply anesthetized used 3.5% chloral hydrate peritoneal injection (10.5 mg/kg) and perfused through the heart with cold PBS and fixed in 4% paraformaldehyde. Serial sections of 5 μm thickness were cut from paraffin embedded tissue blocks and placed onto glass slides. Paraffin sections were deparaffinized and hydrated through a series of graded alcohol. Endogenous peroxidase activity was inactivated with 0.3% hydrogen peroxide. For antigen retrieval, the glass slides were immersed in citrate buffer (0.01 M) at 100 °C for 10 min, and refrigerated at room temperature. Tissue slices were incubated with primary antibodies against RAGE (1:50), HO-1, HQO-1, Nrf2, TNF-α and ICAM-1 (1:200) respectively at 4 °C overnight. Tissue sections were then washed in PBS (0.1 M,pH7.4,3 × 5 min) and transferred for incubation with an appropriate secondary antibody (1:200). The slices were visualized by microscopy using a digital image‑capture system (ECLIPSE-TC, NiKon Microsystems, Japan).

### Western blot analysis

The rats were deeply anesthetized used 3.5% chloral hydrate injection (10.5 mg/kg) and perfused through the heart with cold PBS. Then protein was extracted and concentration was determined. Samples were electrophoresed in SDS/PAGE gels and transferred onto a PVDF membrane and incubated overnight at 4 °C with appropriate primary antibodies against RAGE (1:50), HO-1, HQO-1, Nrf2, MMP9, COX-2, TNF-α and ICAM-1 (1:200). After incubation with horseradish peroxides’ conjugated secondary antibodies (1:1000) for 1 h at room temperature, the blots were developed with chemiluminescence reagent using an ECL kit (Millipore, USA).

### Statistical analysis

The data were processed with one-way analysis of variance (ANOVA), followed by Dunnett’s multiple comparison tests. The results are expressed as the mean ± standard deviation. SPSS software version 17.0 was used for statistical analysis, *P*-values < 0.05 was considered to be statistically significant.

## Results

### SLI treatment has no influence on blood glucose value 24 h after MCAO in the T1DM rats

**Fig. 2:**
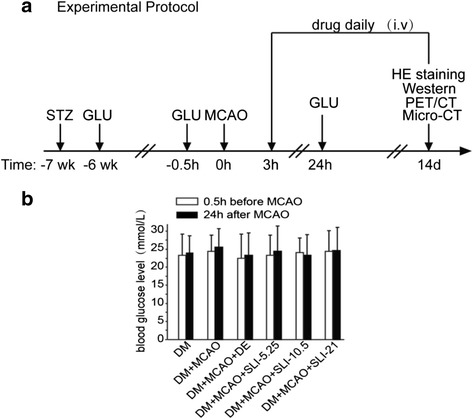
SLI treatment has no influence on blood glucose value 24 h after MCAO in T1DM rats. **a** Schematic of the experiment protocol. **b** SLI treatment have no influence on blood glucose value 24 h after MCAO in T1DM rats

At 7 weeks after STZ injection, the range of blood glucose concentration was about 15-30 mmol/L, which reflected that rats were affected by type I diabetes mellitus. Blood glucose concentrations were monitored at before MCAO and at 24 h after MCAO in T1DM-rats, which remained stable and had no significant difference (Fig. 2).

### SLI treatment significantly decreased neural cell injury in the ischemic penumbra of cortex in the T1DM + MCAO rats

**Fig. 3:**
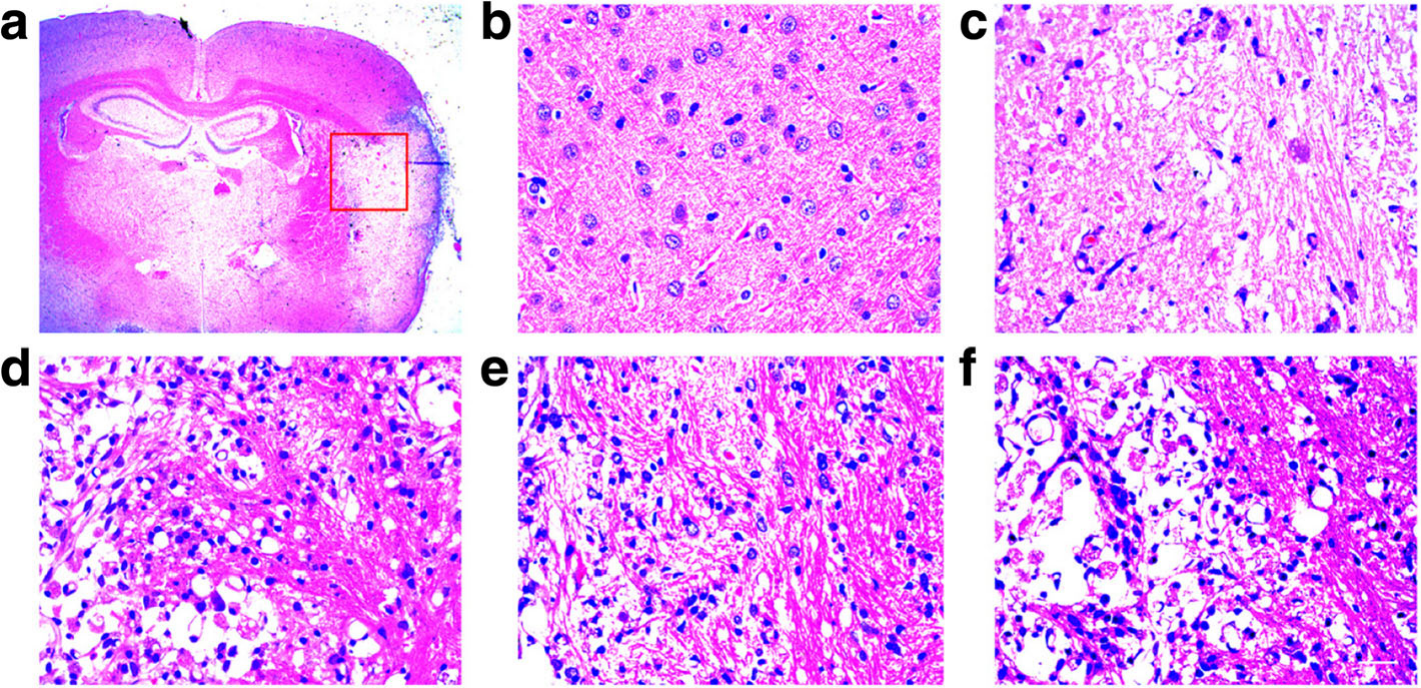
The effect of SLI treatment on neural cell injury in the ischemic penumbra of cortex in the T1DM + MCAO rats. **a** Schematic showing examples of the areas (rad squares); **b** DM-sham, **c** DM-MCAO, **d** DM-MCAO + DE, **e** DM-MCAO + SLI 10.5 mg/kg, **f** DM-MCAO + SLI 21 mg/kg. *N* = 3. Scale bars = 25 μm

Neuronal morphology of the rat brain was observed 14d after MCAO in TIDM rats by HE staining (Fig. 3). The neuronal cells in the DM-sham group were arranged regularly, and the structures of neurons were clear with round, large and regular nuclei. After DM-MCAO, most cells were arranged disorderly, with pyknotic or severely shrunken nuclei in the penumbral region. The morphology changes in the DM-MCAO + ED group were gentler than in the DM-MCAO group. Compared with the DM-MCAO group, less cellular damage was observed, and some neurons showed slightly shrunken perikarya and nuclei in the DM-MCAO + SLI10.5 mg/kg group and the DM-MCAO + SLI 21 mg/kg group.

### SLI treatment significantly increased number of brain microvasculature in ipsilateral in the T1DM + MCAO rats

**Fig. 4:**
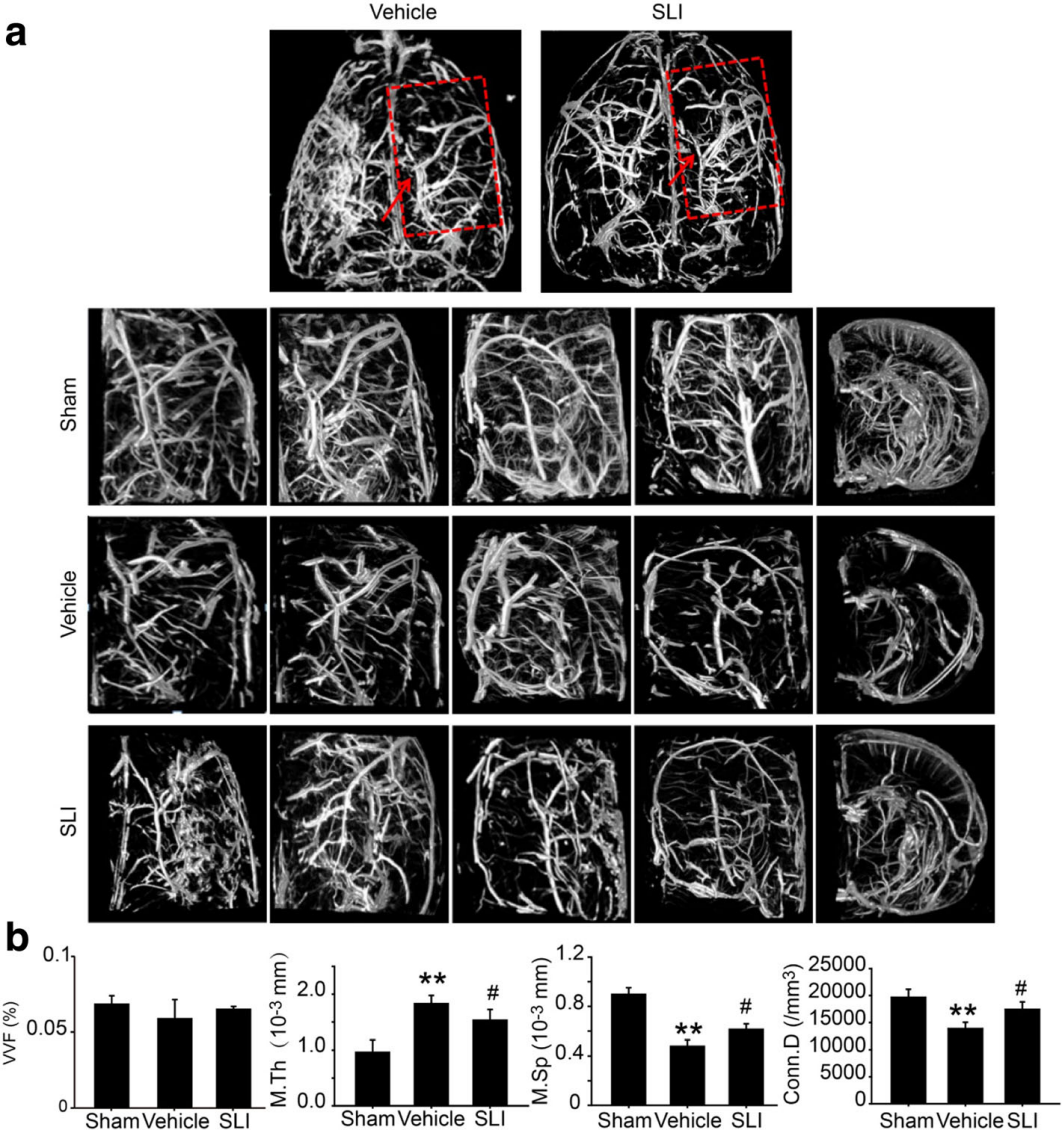
SLI treatment significantly increased number of brain microvasculature in ipsilateral in T1DM + MCAO rats. 3D images of the rat brain microvasculature were obtained using Micro-CT. **a** 3D images of the rat brain microvasculature 14 days after SLI (21 mg/kg) treatment in T1DM + MCAO rats. **b** 3D images of brain microvasculature quantitative analysis. *N* = 4. **p < 0.01 vs DM, #*p* < 0.05 vs DM + MCAO

To investigate the influence of SLI on cerebral blood vessel at 14 days after MCAO in TIDM rats, the number of brain microvasculature was evaluated by Micro-CT. The result showed that SLI (21 mg/kg) treatment apparently increased number of brain microvasculature in ipsilateral in TIDM + MCAO rats (Fig. 4a). As shown in Fig. 4b, SLI treatment significantly increased the M.Sp, Conn.D and decrease M.Th in TIDM + MCAO rats.

**Fig. 5:**
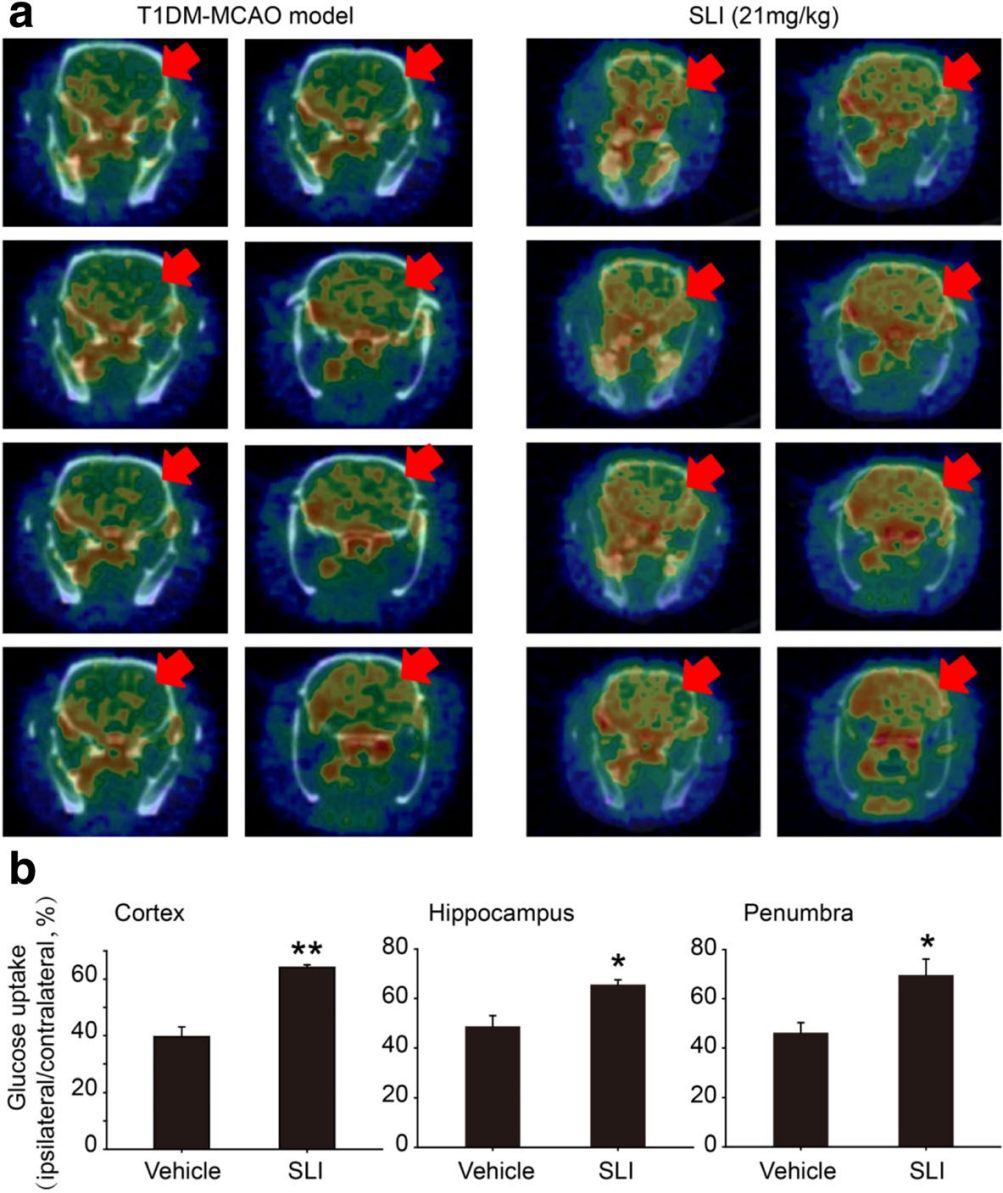
SLI treatment significantly increased glucose uptake at cortex, hippocampus and penumbra in the T1DM + MCAO rats after 14 days. **a** PET-CT images of the rat brain at 14 days post-surgery after SLI treatment. **b** PET-CT images of glucose uptake quantitative analysis. *N* = 4. **p < 0.01, *p < 0.05 vs DM + MCAO

**Fig. 6:**
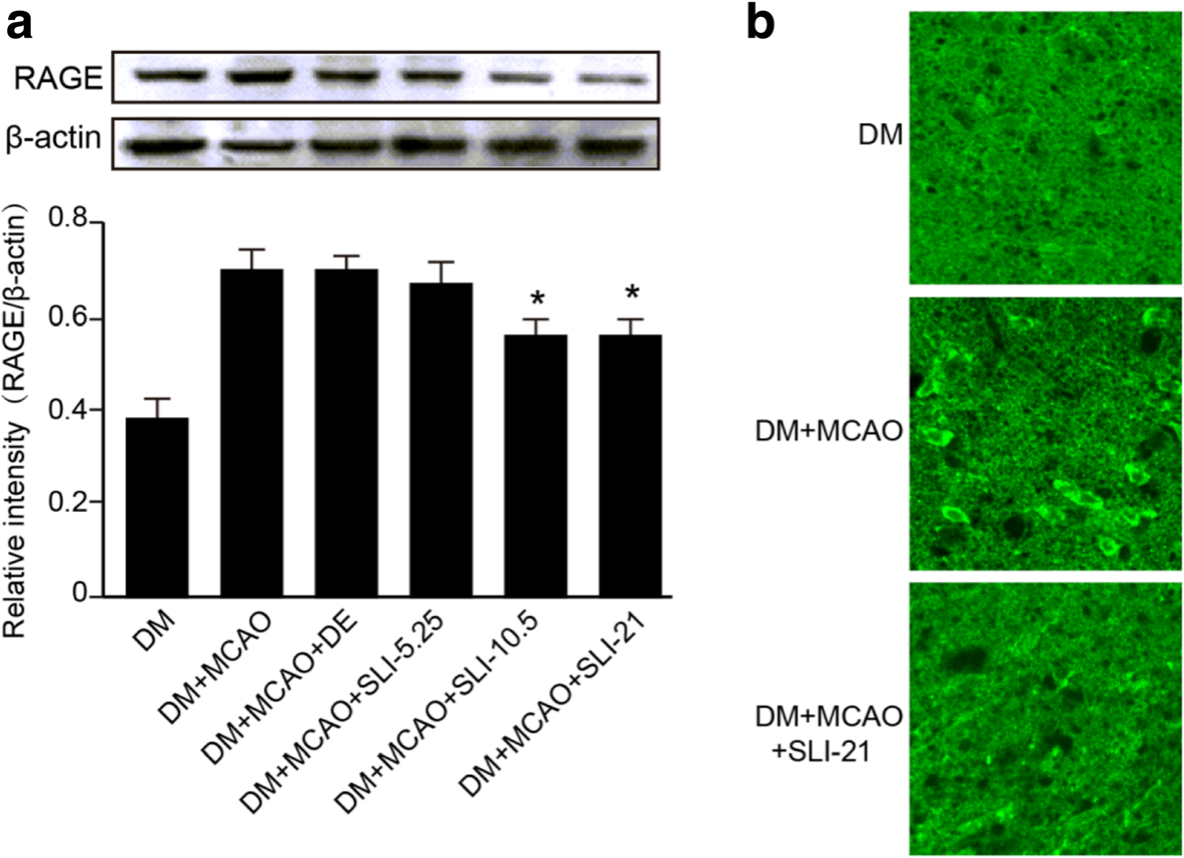
SLI treatment significantly decreased RAGE expression in T1DM + MCAO rats. **a** Western blot assay and quantitative data for RAGE after SLI treatment in the T1DM + MCAO rats. **b** An immunofluorescent analysis for RAGE after SLI (21 mg/kg) treatment in T1DM + MCAO rats. *N* = 6. *p < 0.05, **p < 0.01 vs DM + MCAO

**Fig. 7:**
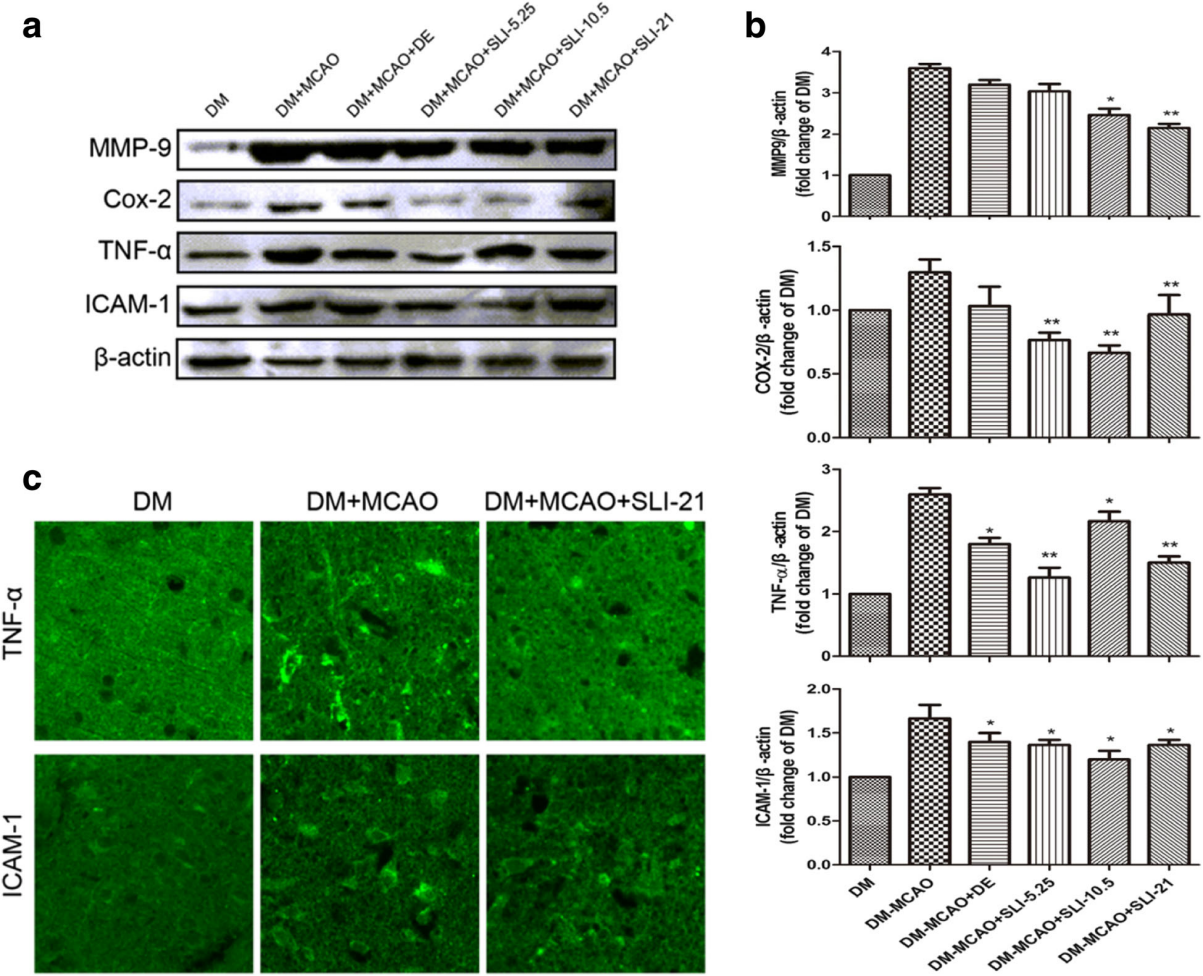
SLI treatment significantly decreased MMP9 and inflammatory factors expression in T1DM + MCAO rats. **a**, **b** MMP9,COX-2,TNF-$\boldsymbol{\upalpha}$ and ICAM-1 western blot assay and quantification data after SLI treatment in the T1DM + MCAO rats. **c** TNF-$\boldsymbol{\upalpha}$ and ICAM-1 immunofluorescent data after SLI (21 mg/kg) treatment in T1DM + MCAO rats. *N* = 6. *p < 0.05, **p < 0.01 vs DM + MCAO

**Fig. 8:**
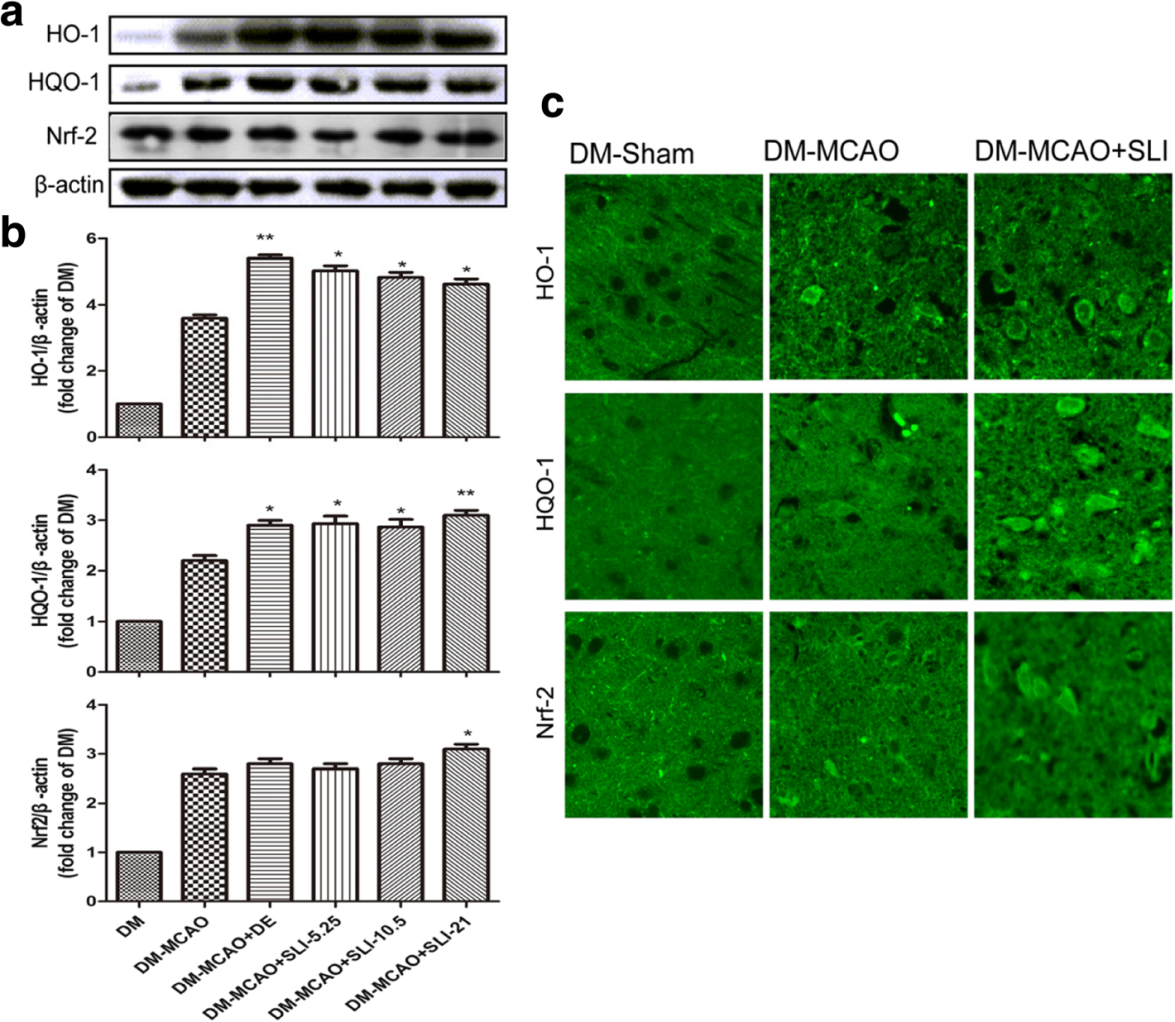
SLI treatment significantly increased HO-1, HQO-1 and Nrf-2 expression in T1DM + MCAO rats. **a**, **b** Western blot and quantitative analysis of the level of HO-1, HQO-1 and Nrf-2 expression after SLI treatment in T1DM + MCAO rats. **c** An immunofluorescent analysis data of HO-1, HQO-1 and Nrf-2 expression after SLI (21 mg/kg) treatment in T1DM + MCAO rats. *N* = 6. *p < 0.05, **p < 0.01 vs DM + MCAO

### SLI treatment significantly increased glucose uptake in cortex, hippocampus and penumbra in the T1DM + MCAO rats

In order to evaluate effect on glucose uptake of SLI treatment at 14 days post-surgery in T1DM + MCAO rats, PET-CT images of glucose uptake was performed. Figure 5 showed that SLI (21 mg/kg) treatment significantly increased glucose uptake in cortex, hippocampus and penumbra in the T1DM + MCAO rats.

### SLI treatment significantly decreased RAGE expression in the T1DM + MCAO rats

To test whether SLI treatment of stroke in T1DM rats regulates inflammatory factor RAGE expressions, an immunofluorescent and a western blot analysis for RAGE expression were performed. In Fig. 6, RAGE expression was significantly decreased after SLI treatment in T1DM + MCAO rats and immunofluorescent result confirmed this result.

### SLI treatment significantly decreased MMP9 and inflammatory factors expression in the T1DM + MCAO rats

To understand the underlying mechanisms of SLI treatment functional improvement in T1DM-MCAO rats, MMP9 and inflammatory factors expressions were quantified in the ischemic zone. As shown in Fig. 7, MMP9, COX-2, TNF-$\boldsymbol{\upalpha}$ and ICAM-1 expression was significantly decreased after SLI treatment in T1DM + MCAO rats.

### SLI treatment significantly increased HO-1, HQO-1 and Nrf-2 expression in the T1DM + MCAO rats

To clarify whether the Nrf2/HO-1 pathway was involved in the neuroprotection of SLI, the expression level of proteins related to Nrf2/HO-1 pathway after SLI treatment in T1DM + MCAO rats were carefully detected by Western blots and Immunofluorescent. The results showed that HO-1, HQO-1 and Nrf-2 expression was significantly increased after SLI treatment in T1DM + MCAO rats. These results were confirmed by immunofluorescent test (Fig. 8).

## Discussion

Diabetes mellitus (DM) is a leading health concern associated with both microvascular and macrovascular diseases and leads to threefold to fourfold higher risk of experiencing ischemic stroke [20, 21]. DM patients face greater residual neurological and functional disability and DM is associated with more severe strokes and poor recovery compared with nondiabetic individuals [22]. Clinical and experimental studies have shown that DM not only increases the risk and recurrence of ischemic stroke, but also leads to poorer outcomes and increases fatality rates after stroke [21, 23, 24]. In this study, we have demonstrated that SLI treatment significantly increased the number of brain microvasculature and improved glucose uptake in cortex, hippocampus and penumbra in the T1DM + MCAO rats through multiple mechanisms of action, including decreasing the expression of RAGE, MMP9 and inflammatory factors (COX-2, TNF-α and ICAM-1), and increasing the expression of HQ-1, HQO-1 and Nrf-2 in T1DM + MCAO rat.

Salvianolate lyophilized injection (SLI) composes of the Salvia miltiorrhiza aqueous extraction (mainly including salvianolic acid B, salvianolic acid E, lithospermic acid and rosmarinic acid). Salvianolic acid B is the most abundant and bioactive compound of Salvianolate lyophilized injection. Many studies have demonstrated that salvianolic acid B exerts various pharmacological activities, such as anti-apoptosis, anti-inflammation, anti-diabetes, anti-oxidation, promotion of cellular proliferation, differentiation and bone formation, anti-tumor and preservation of normal cell functions [25]. Salvianolic acid B confers neuroprotection via anti-inflammatory and anti-oxidative effects and is attenuate VCAM-1 and ICAM-1 in expression TNF-α-treated human aortic endothelial cells HAECs [26, 27]. Likewise, salvianolic acid B protects blood-brain barrier in rats after cerebral ischemia-reperfusion though inhibiting the MAPK pathway [28]. Many bioactivities of rosmarinic acid have been reported, such as anti-liver fibrosis, antisepsis and anti-diabetic nephropathy [29, 30]. Rosmarinic acid has significant neuroprotective effects during cerebral I/R injury, such as attenuation of BBB breakdown, a decrease of infarct volume and reduction of HMGB1 expression in ischemic brain tissue. Lithospermic acid, a similar structure with salvianolic acid B, is reported to have anti-oxidative activity, restore liver functions and inhibit apoptosis against CCL4 toxicity [31]. All these reports show that the active components are effective for the treatment of stroke in cerebral ischemia model without type 1 diabetic. Whereas, we used a model of transient focal ischemia to observe whether SLI could influence brain injury following ischemia-reperfusion in type 1 diabetic rat.

Though there is much evidence to suggest that the active components of this herb are effective for the treatment of stroke, the effective of these active components in cerebral ischemia in type 1 diabetic rat isn't reported. In this study, we aimed to investigate the effects of SLI in cerebral ischemia in type 1 diabetic rat. Based on these bioactive compounds, we suppose that SLI could prevent against ischemic stroke with diabetes mellitus through various ways, although our present study just explored one signaling pathway.

We have previously demonstrated that SLI apparently reduced the infarct volume by Magnetic Resonance Imaging (MRI) and TTC staining in T1DM + MCAO rats [4]. Therefore, in the present study, we focused on the mechanism of SLI treatment T1DM rats on the recovered progression of cerebral ischemia. Over the past thirty years, micro-CT imaging has been widely used for the 3D-morphological characterization, examination and quantification, including the brain [32]. High quality brain micro-CT provides both structural and functional information about the murine following ischemia and reperfusion. Positron emission tomography (PET) is increasingly used for quantitative perfusion imaging in ischemic disease and has been used for microglial neuro-inflammation following stroke in human and animal models [33–36]. These methods are more accurate in evaluating brain function compared with triphenyltetrazolium chloride staining and the neurological deficit score. Here we explore adapting Micro-CT and PET-CT to image brain microvasculature and glucose uptake in ipsilateral in T1DM + MCAO rats and found that SLI treatment significantly improved brain function in these rats.

RAGE gene is expressed at low levels in the majority of normal human tissues [37, 38]. But the enhanced expression of RAGE is observed in diabetic vasculature and other inflammatory diseases. Matrix metalloproteinase MMP-9, which is an important collagenase of the MMP family, has previously been widely studied in acute cerebral ischemia. MMPs are important in the breakdown of the BBB and cerebral edema, and pathophysiologic processes involving angiogenesis following cerebral ischemia. Stroke is a complex disorder characterized by variable gene expression and intermediate phenotypes, and the inflammation of artery plays an important regulatory role in progression of stroke [39]. COX-2 is highly expressed in perifocal striatal neurons, blood vessels, and endothelial cells after cerebral ischemia. Likewise, COX-2 is increased levels of inflammatory cytokines [40]. It is associated with pathogenesis that occurs both early and late during cerebral ischemia, and COX-2 is overexpressed after ischemic stroke and contributes to the production of neurotoxic and to oxidative stress associated with inflammation and ischemic brain damage [41]. The increased expression of TNF-α after ischemia may up-regulate adhesion molecules such as ICAM-1, which can damage the endothelium and BBB, Overexpression of TNF-α exacerbates ischemic cerebral damage, whereas their inhibition decreases cerebral edema and infarct volume [42]. Our report shows that SLI treatment prevent brain injury after ischemic stroke by decreasing the expression of RAGE, MMP9 and inflammatory factors (COX-2, TNF-α and ICAM-1) in T1DM + MCAO rats.

Nuclear factor erythroid 2-related factor 2 (Nrf2) plays an important regulatory role in the protection of cells against oxidative stress [43]. The Nrf2/HO-1 pathway has been shown to play an important neuroprotective role in brain injury after ischemic stroke. Recently, Shah, Z. A. and Alfieri had explored the therapeutic potential of targeting the Nrf2/HO-1 pathway in brain injury after ischemic stroke [9, 44]. In view of the importance of the Nrf2 defense pathway in neuroprotection, we examined the possibility effects of SLI treatment on the Nrf2/HO-1 pathway. Our results showed that SLI treatment significantly increased HQ-1, HQO-1 and Nrf-2 expression in T1DM + MCAO rats. In Fig. 8a, the levels of Nrf2 in each group are similar. In order to better understand this phenomenon, we retrieved some references that showed the potential mechanism of edaravone for the treatment of focal cerebral ischemia and reperfusion injury. These findings indicate that edaravone treats cerebral ischemia-reperfusion injury in rats via repressing HIF-1α signaling pathway [45] or through a Bax/Bcl-2 dependent anti-apoptotic mechanism [46], which are not consistent with the mechanism of Nrf2. Thus, the levels of Nrf2 are similar between DM + MCAO and DM + MCAO + DE group. Because Salvianolate lyophilized injection (SLI) mainly includes many active components such as alvianolic acid B, salvianolic acid E, lithospermic acid and rosmarinic acid, and the mechanism of SLI for the treatment of stroke is multi-targeted. Meanwhile, Nrf2 is one of the mechanisms of SLI for the treatment of stroke. The result has the obvious difference with the increasing dose of SLI. Therefore, In Fig. 8, we can observe the obvious difference at the dose of 21 mg/kg.

Although we demonstrated the cerebral protection of SLI in T1DM + MCAO rats, the detailed mechanisms of SLI on Nrf2/HO-1 signaling pathways need to future study. In addition, how anti-inflammation program and angiogenesis are regulated by SLI is worthwhile to study in the future.

## Conclusion

In summary, our study demonstrates that SLI confers protection against T1DM + MCAO induced brain injury, at least partly through decreasing RAGE, MMP9 and inflammatory factors expression and up-regulating Nrf2/HO-1 antioxidant pathway. These findings may contribute to the better understanding of the molecular mechanisms involved in cerebral protection of SLI and provide novel insights into future therapeutic strategy for ischemic stroke.

## Abbreviations

AGEs: Advanced Glycation End Products
ANOVA: One-way analysis of variance
CCA: Common carotid artery
Conn.D: Connectivity Density
COX-2: Cyclo-oxyge-nase-2
DE: Edaravone
ECA: External carotid artery
HO-1: Heme-oxygenase-1
HQO-1: NAD(P)H quinine oxidoreductase
ICA: Internal carotid artery
ICAM1: Intercellular cell adhesion molecule-1
LA: Lithospermic acid
M,Th: Mean Vascular Thickness
M.Sp: Mean Vascular Separation
MCAO: Embolic middle cerebral artery occlusion
MMP9: Matrix metallopeptidases 9
Nrf2: Nuclear factor erythroid 2-related factor 2
RA: Rosmarinic acid
SalB: Salvianolic acid B
SLI: Salvianolate lyophilized injection
STZ: Streptozotocin
T1DM: Type 1 diabetic
TCM: Traditional Chinese Medicine
TNF-$\boldsymbol{\upalpha}$: Tumor necrosis factor-$\boldsymbol{\upalpha}$
VVF: Vascular Volume Fraction
